# The immunology connection—my first T cell receptor structure projects

**DOI:** 10.1007/s13238-014-0091-7

**Published:** 2014-08-05

**Authors:** Jia-huai Wang

**Affiliations:** Dana-Farber Cancer Institute, Harvard Medical School, Boston, MA 02115 USA

Ellis Reinherz and I were riding in a luxury Boston Coach to Long Island in New York State driven by a professional driver. This was not a vacation trip to one of the best leisure places in the United States. We were heading toward the National Laboratory at Brookhaven, where a powerful X-ray source is available in a facility called a synchrotron. The aim of our trip was to use a strong X-ray to shoot the crystals of the T cell receptor (TCR) we had just managed to grow after considerable effort for three-dimensional structure determination. In 1996, this was one of the hottest projects in structural biology as well as in immunology.

I got acquainted with Ellis when I was a visiting scholar from China working in Stephen Harrison’s lab at Harvard for another high-profiled project: to solve the structure of CD4. CD4 had long been known as the co-receptor of TCR, playing a key role in the immune system since its description in 1979 (Reinherz et al., 1979). But in 1988 to determine the structure of CD4 had a much more urgent goal behind. In the early 1980s, HIV was discovered as the cause of the life-threatening acquired immunodeficiency syndrome (AIDS), and soon CD4 was identified as the primary receptor of HIV. The virus uses CD4 to open the door for invading the T cell, eventually destroying the whole immune system, hence the name of AIDS. Steve and the late Don Wiley initiated and directed this extremely important structure project with a clear goal in mind: To solve the structure of CD4, which might be potentially the first step to ultimately stop the HIV pandemic. CD4 as a transmembrane receptor has four immunoglobulin (Ig) like domains on the cell surface. Steve was seeking all possible sources to collect the protein sample with different domain constituents for crystallization. Ellis was one of the sources. Ellis’s lab was able to express protein composed of the N-terminal two domains of CD4. By then it was already known that the very N-terminal domain of CD4 is solely responsible for virus binding. We were hoping that a two-domain construct would be sufficient for this purpose. My wife was assigned to grow crystals. She often was handed over the protein sample by Ellis’s technician in Harvard Square, a few miles away from his lab near the downtown Boston! The protein Ellis produced did yield excellent crystals. It was a truly exciting moment when for the first time, I watched the high-resolution diffraction pattern of the CD4 crystal on the detector using the home source X-ray machine. I knew the structure could be on the horizon. At that time, there was no fancy software capable of computing the phasing, electron density modification and automatic model building, poly-peptide tracing on the density map from the experimental phasing was always a challenge. After I obtained the multiple-isomorphous replacement map, it was quite difficult to interpret the noisy map. Using a homologous immunoglobulin domain as a model, Steve himself did the N-terminal domain fitting on the graphics machine to make the breakthrough. We then successfully traced the second domain on a so-called mini-map, which was a stacked plastic sheet with electron density maps contoured in an old-fashion way to determine the proteins’ backbone structure. The international competition to determine this structure was unprecedented. My friend Jack Strominger, a veteran immunologist at Harvard, was once teasing me by saying that “Jia-huai, there are at least 15 labs in the world working on the same project!” In the end, two labs advanced far enough to reach the agreement of coordinating the publication together (Ryu et al., 1990; Wang et al., 1990). The other lab was led by Wayne Hendrickson at Columbia University. The news on the two *Nature* articles was in the New York Times in 1990. It is interesting to add that it was not until ten years later in 2001 when I was already on the faculty of Dana-Farber Cancer Institute and Harvard Medical School that Ellis and I published another structure of CD4 (Wang et al., 2001). This time it was in complex with the class II MHC molecule, in which we showed clearly how HIV uses a similar strategy to outcompete CD4’s physiological binding partner MHC to grab CD4 for the invasion. That effort complemented the first class II MHC-restricted TCR in complex with pMHCII to be described by us in 1999 (Reinherz et al., 1999). In those two structures, the complexes included the same pMHCII, allowing us to infer and create a molecular model of the TCR-pMHC-CD4 tri-molecular complex solved by our colleague Roy Marriuzza a decade later (Yin et al., 2012).

On the 5-hour trip to Long Island, Ellis and I had to carefully hold, by turn, the styrofoam box in which the crystallization trays were carefully packed. The tiny crystals grew in 2 microliter droplets in 24-well trays. We just could not risk any damage to these extremely valuable but fragile crystals, so we handheld the box all the way to avoid any significant shaking when the coach was bumping on the road. Producing these soluble TCR molecules for crystallization was not an easy job, and still isn’t almost twenty years later. In Ellis’s lab, a team led by two highly skillful molecular biologists and protein chemists tried every available technique to clone, express and purify several kinds of TCR. One of the challenging issues to produce soluble TCR molecules is that like many other cell surface receptors, TCR molecules are glycosylated. Bacterium expression systems do not have this post-translational modification mechanism. Without glycosylation, the bacteria-expressed protein may be less soluble and tends to aggregate. At that time knowledge about the role of glycans was very limited. We just had no idea whether the glycan on the TCR molecule was of biological significance. One of the postdoctoral fellows in the lab turned to the mammalian system, the CHO (Chinese Hamster Ovary) cell to express the protein, because these cells can produce glycoproteins. Then another headache struck. Glycosylation is notoriously heterogeneous. In order to grow crystals of diffraction quality, the prerequisite is to have a chemically homogeneous protein sample. Fortunately, scientists around that time developed an engineered CHO, termed CHO lec^–^, which is able to express homogeneous, mannose-enriched glycoprotein in large quantities (Stanley, 1989). At that point we started to have some ugly-looking crystals of TCR. One more advantage of using the CHO lec^–^ system is that the produced glycoprotein can be treated with some enzymes for deglycosylation. All of a sudden by using the deglycosylated homogenous TCR sample, beautiful crystals appeared. This marked a big step toward our final goal. Using the home source X-ray machine, around 8–10 Å diffraction spots could be seen for these crystals. At that moment I knew a synchrotron trip was inevitable.

Bob Sweet was in charge of the National Synchrotron Light Source (NSLS) beamline X12C at Brookhaven. I actually met Bob personally more than ten years ago when I paid a visit to David Eisenberg’s lab at UCLA in 1980. Bob was David’s postdoctoral fellow then who hosted my visit. We had a very interesting talk on his project. With Bob as a host at NSLS, I felt very comfortable. He was friendly and introduced us to his facility, after Ellis and I safely arrived and most of crystals seemed to survive. On the site also was a group from Albert Einstein College led by Jim Sacchettini, another X-ray crystallographer with whom Ellis had previously established a collaboration. That was the very early days when protein crystallographers just began to freeze protein crystals to collect diffraction data at around −140°C. To protein crystallographers, the synchrotron radiation source was like a double-edged sword. On one hand, the synchrotron X-ray source was so powerful, it had made possible the data collection of those weakly diffracting crystals like TCR. On the other hand, the X-ray was so strong that it quickly damaged these fragile crystals before any complete data set could be finished. To collect a data set at −140°C under cold stream generated from liquid nitrogen was invented early in 1990’s to circumvent the problem. X-ray damage was no longer a headache. Bob had a well set-up facility for all these purposes.

I would never have expected to almost lose all my crystals brought to Brookhaven that day. I thought I was well prepared for the data collection because I had already carried out all the experimental procedures at home in Boston. The well-developed crystals were safely harvested from the tiny droplet into a large well with so-called cryo-protectant solution. These incubated crystals could then be fished up using a small nylon loop supported on a metal cap, and quickly flash frozen by mounting the cap on the diffractometer equipped with cold stream from liquid nitrogen, ready to shoot with X-rays. The problem arose when the cap was mounted on the machine by the magnetic force. On Bob’s machine, the magnet was so strong that the moment a crystal was put on, the force that attracted the cap on the machine shook the crystal off from the nylon loop, no matter how careful I was. We only had three crystals had left. The atmosphere, not the crystals, seemed frozen. Ellis suggested that everyone leave the room. He then turned to me and said: “Jia-huai, I am going to buy lunch for you. Just relaxed”. That did help reduce the tension! I used my right hand to fish crystal, at the same time I used my left hand to help hold the cap more smoothly and set the crystal on. A beautiful diffraction pattern showed up. This was our first 2.8 Å data set of TCR crystal! That would lead to our 1998 published study (Wang et al., 1998).

Like CD4, the TCR structure project was also in a very tense competition. Christopher Garcia in Ian Wilson’s group at Scripps Institute and David Garboczi in Don Wiley’s group at Harvard were far ahead of us in solving the first MHC class I restricted TCR. By the time we were trying to determine the structure by locating the heavy atom (in fact it was selenium that was genetically replacing the sulfur atom in metheonine) sites, these two groups had published their structures of TCR alone and in complex with pMHC (Garboczi et al., 1996; Garcia et al., 1996). We then could use Wilson’s structure to quickly solve ours using molecular replacement and published in *EMBO J* in 1998 (Wang et al., 1998). We also took advantage of being the third group to compare the three TCR/pMHC structures and identified a common binding mode of TCR onto pMHC (Teng et al., 1998). With over several dozen TCR complex structures currently deposited in the Protein Data Bank, this common docking scheme still holds.

The ecto-domains of α and β subunits of TCR are both made up of two Ig-like domains: a variable domain and a constant domain. There are two obvious deviations of the two TCR constant domains from a canonical Ig-like domain. First is the strange constant domain of α subunit. It could hardly be designated as an Ig-like domain, which normally consists of two β sheets packing face-to-face. The TCR α constant domain does have an inward β sheet facing the constant domain of β subunit. But the outward face is no longer a β sheet. One of the strands curves into a helical conformation, whereas the other two strands just loosely hang around. When our structure got published, Ian Wilson told me that he felt so much relieved to see that our α constant domain was just like theirs since the Wiley group could not trace this outside sheet in their structure. We still have no idea even today why this domain appears so poorly structured. This became even more of a mystery when Jamie Rossjohn published his pre-TCR structure (Pang et al., 2010), a TCR precursor that only exists during T cell development in the thymus. The pre-TCR molecule does not have the variable domain of its α subunit. However its constant domain assumes a perfect canonical Ig-like domain!

As a structural biologist, I was also fascinated by the second deviation of TCR structure from the normal Ig-like domain, the unusually long loop between the F strand and G strand, stretching out of the constant domain of the β subunit. I noticed that this was a common feature shared in the other two published structures. We now know that this is a very conserved structural feature of αβ TCR molecules and evolved with the molecular speciation of CD3γ and CD3δ from a common precursor molecule several hundred million years ago (Kim et al., 2010). Remarkably, in the center of the FG-loop lies a bulky tryptophan amino acid residue, around which exquisite hydrogen bonds and hydrophobic interactions make the loop a relatively rigid entity (Wang et al., 1998). My structural observation immediately caught Ellis’s attention. He thought this loop might bear an important function in the TCR signaling process by rigidifying this subunit and in turn pressing on the heterodimeric CD3 signaling molecules that collectively comprise the TCR complex. Sixteen years have passed since this early structural observation and we have never stopped investigating the role that this unique FG-loop might play in TCR signaling. The issue has frequently been the discussing topics in our weekly lunch meeting. Our most recent collaborative efforts are to use single molecule technique to explore the function of this key structural element.

“This is my fourteen-year dream”, said Ellis to me on the way back from that Brookhaven trip. He was referring to the day he and his colleagues at Dana-Farber Cancer Institute discovered the human TCR in the early 1980s. We both were so excited and kept chatting about how to proceed that we didn’t notice the downpour going on outside the coach. We finally realized that the poor driver missed our exit to Boston on the highway and we were on the way to Maine! The trip took more than two hours longer it should be, yet the sweet memory is still kept vividly present in my brain ever since.
